# AK-DL: A Shallow Neural Network Model for Diagnosing Actinic Keratosis with Better Performance than Deep Neural Networks

**DOI:** 10.3390/diagnostics10040217

**Published:** 2020-04-13

**Authors:** Liyang Wang, Angxuan Chen, Yan Zhang, Xiaoya Wang, Yu Zhang, Qun Shen, Yong Xue

**Affiliations:** 1 Beijing Advanced Innovation Center for Food Nutrition and Human Health, Key Laboratory of Plant Protein and Grain Processing, National Engineering and Technology Research Center for Fruits and Vegetables, College of Food Science and Nutritional Engineering, China Agricultural University, Beijing 100083, China; 18259800533@163.com (L.W.); 15384665858@163.com (X.W.); shenqun@cau.edu.cn (Q.S.); 2 College of Information and Electrical Engineering, China Agricultural University, Beijing 100083, China; angxuan.chen@foxmail.com (A.C.); edison_yan@icloud.com (Y.Z.); 18810760793@163.com (Y.Z.)

**Keywords:** actinic keratosis, AK-DL, intelligent diagnostic app, mainstream deep model

## Abstract

Actinic keratosis (AK) is one of the most common precancerous skin lesions, which is easily confused with benign keratosis (BK). At present, the diagnosis of AK mainly depends on histopathological examination, and ignorance can easily occur in the early stage, thus missing the opportunity for treatment. In this study, we designed a shallow convolutional neural network (CNN) named actinic keratosis deep learning (AK-DL) and further developed an intelligent diagnostic system for AK based on the iOS platform. After data preprocessing, the AK-DL model was trained and tested with AK and BK images from dataset HAM10000. We further compared it with mainstream deep CNN models, such as AlexNet, GoogLeNet, and ResNet, as well as traditional medical image processing algorithms. Our results showed that the performance of AK-DL was better than the mainstream deep CNN models and traditional medical image processing algorithms based on the AK dataset. The recognition accuracy of AK-DL was 0.925, the area under the receiver operating characteristic curve (AUC) was 0.887, and the training time was only 123.0 s. An iOS app of intelligent diagnostic system was developed based on the AK-DL model for accurate and automatic diagnosis of AK. Our results indicate that it is better to employ a shallow CNN in the recognition of AK.

## 1. Introduction

Actinic keratosis (AK), also called senile keratosis, is usually caused by long-term exposure to sunlight or ionizing radiation [1,2]. It is one of the most common precancerous skin lesions and presents as reddish brown or yellow macules or small plaques with clear boundary. The theory of field cancerization postulates that the skin encircling AK is also at increased risk of possible malignant transformation because it has suffered from the same chronic ultraviolet light [3]. Early medical diagnosis and treatment are believed to delay the development of AK and the cancerization of the skin surrounding it [3,4,5]. However, AK can be easily confused with benign keratosis (BK), such as seborrheic keratosis (SK). At present, the clinical diagnosis is mainly based on histopathological examination with a lengthy and complicated process [6,7,8,9]. 

With the development of computer vision, image recognition based on deep learning is increasingly being used in medical diagnostics [10,11,12,13,14,15], including cancer identification, histocyte classification, tumor image segmentation, etc. Kawahara et al. [16] used convolution neural network (CNN) early on to classify dermatologic images and achieved good results. Kaur et al. [17] demonstrated that the classification accuracy of hybrid deep learning method could reach up to 82.0% among six kinds of skin images, which was better than the traditional CNN models. Romero-Lopez et al. [18] exploited VGGNet to classify benign and malignant types of skin cancer and achieved a classification accuracy of 78.66% based on the International Skin Imaging Collaboration (ISIC) dataset. Harangi et al. [19] incorporated softmax layer outputs of four different neural architectures for intelligent diagnosis of melanoma and eventually found that this method was superior to single neural network. Generally, research on the use of machine learning to identify skin diseases is dominated by mainstream deep CNNs, which are characterized by long duration of training and high requirement of arithmetic facility. Recent studies have found that the performance of shallow CNNs are not worse, but even better, than deep CNNs in some cases [20,21]. However, such results have not been found in dermatology, especially in recognition of AK. The excellent image identification of actinic keratosis based on a shallow CNN should have high accuracy, high efficiency, and low requirement for operating equipment so that it can improve the efficiency of clinical diagnosis and facilitate self-monitoring and prevention of skin cancer.

In this study, we designed a new CNN model named actinic keratosis deep learning (AK-DL), which includes two convolutional layers and a max pooling layer, to recognize actinic keratosis. We then compared it with the current mainstream deep CNNs of skin mirror diagnosis, such as AlexNet, GoogLeNet V1, and ResNet-50, as well as traditional medical image processing methods. Finally, an iOS app of intelligent diagnostic system was developed based on the AK-DL model.

## 2. Materials and Methods 

### 2.1. Data Acquisition

A public dataset HAM10000 (collected by the following two departments over a period of 20 years: the Department of Dermatology at the Medical University of Vienna, Austria, and the skin cancer practice of Cliff Rosendahl in Queensland, Australia) from the ISIC dataset was employed [22], consisting of 100,015 skin images belonging to 7 common skin diseases. It is worth noting that each mirror in this dataset has been strictly confirmed by dermatologists. A part of the data was used in our study, including 327 skin images diagnosed as AK and 1099 skin images diagnosed as BK (Table S1). To our knowledge, there has been no study using keratosis images alone from the ISIC dataset.

### 2.2. Data Preprocessing

Preprocessing, an indispensable step in computer vision, transforms the initial image into a form that can be computerized. The operations of preprocessing in this study included data augmentation, image enhancement, and color space conversion.

Due to the imbalance of categories in this dataset, the data was augmented to improve the robustness of the model and enhance its generalization ability. The methods of data augmentation usually contain image rotation, image flip, random cropping, adding noise, etc. Considering the fact that the number of AK images was limited, the images were rotated 90 degrees, horizontally flipped, vertically flipped, and 1308 derived samples were finally produced. Data augmentation can not only avoid the poor training results caused by unbalanced data but also prevent overfitting caused by a small dataset.

**Figure 1:**
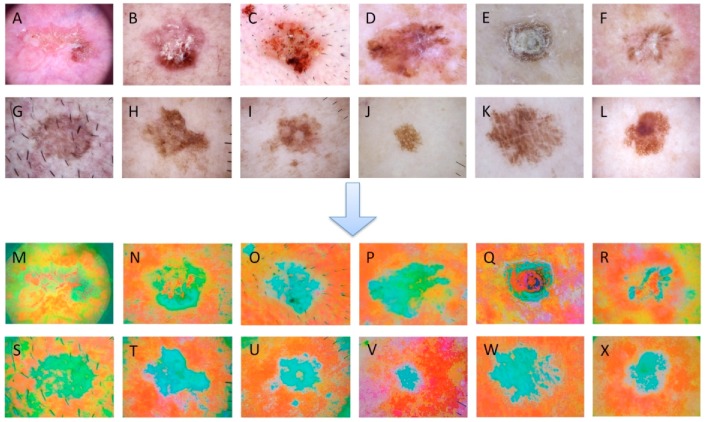
Comparisons of skin images before and after preprocessing. (**A**–**F**) Actinic keratosis (AK) images before preprocessing, (**G**–**L**) benign keratosis (BK) images before preprocessing, (**M**–**R**) actinic keratosis images after preprocessing, (**S**–**X**) benign keratosis images after preprocessing.

To improve the image quality and easily identify key features, images are often enhanced by several methods, such as histogram equalization, contrast enhancement, Gamma correction, etc. In our study, histogram equalization was performed on the amplified images to highlight the texture region of keratosis. Furthermore, the color space of images was converted into Lab space from RGB space for the expansion of color gamut. Lab space has a wider color gamut than RGB and CMYK and is thus a great compensation for the uneven distribution of RGB and CMYK color modes. Additionally, Lab space separates brightness from color; the L channel has no color, while the a and b channels have color, so the adjustment operation is simple and easy for computer processing. The preprocessing of images is shown in Figure 1.

### 2.3. Models Building

Since Lecun et al. [23] first proposed the classic CNN model LeNet-5 in 1999, more and more mainstream deep learning algorithms have been applied in the field of medical images. AlexNet appeared in 2012, followed by VGG, GoogLeNet, ResNet, and other deep CNNs. In this study, we investigated a new deep learning model named AK-DL and compared it with the current mainstream algorithms of medical diagnosis (AlexNet, GoogLeNet, and ResNet). All building and training processes of the models were carried out in Windows 10 operating system with CPU Intel Core I7-6700HQ (3.5 GHz, 4 GB memory).

#### 2.3.1. AK-DL Model

As a classic CNN architecture, the input layer size of the LeNet-5 model is 28 × 28 × 1. The input image is handwritten numeral, and its texture information is simple. However, the texture information of the keratosis image in the present study was relatively complex, so the input layer size was modified to 64 × 64 × 3. The aim of the convolutional layer is to extract different characteristics of the input, and two convolutional layers were set in the AK-DL model. The core parameters of the convolution operation were as follows: the size of the convolution kernel was set as 5 × 5, the convolution stride length was set as 1, and the padding was set as 2. The number of types on the two convolutional layers were set as 6 and 16, respectively. The pooling layer, which is mainly used for feature dimensionality reduction, can reduce the number of parameters, prevent overfitting, and improve the fault tolerance of the model. In this research, a max pooling layer was adopted after the first convolutional layer to construct a neural network, and a 2 × 2 sampling window was used to control the reduction rate of the feature map. Usually, the fully connected layer is used to highly purify the features extracted by the convolutional layer, and it can thus play an important role in mapping the distributed feature with representation of the sample space. In our research, a fully connected layer after the last convolutional layer was added in the AK-DL model. 

The traditional shallow CNN model has a certain shortcoming in generalization ability, resulting in overfitting in the case of insufficient data. Considering the relatively small amount of keratosis images, the dropout layer was introduced following the fully connected layer to prevent overfitting. During training, dropout can discard some neurons in the hidden layer with a certain probability, resulting in changes of the network structure. The whole process of dropout is equivalent to taking the average value of multiple different neural networks. In other words, different networks have different overfitting, and some “inverse” overfitting can cancel each other out to reduce overall overfitting; to some extent, it can also reduce the complex coadaptation among neurons to achieve regularization effect. After several tests, it was found that dropout was performed the best when set as 0.5.

**Figure 2:**
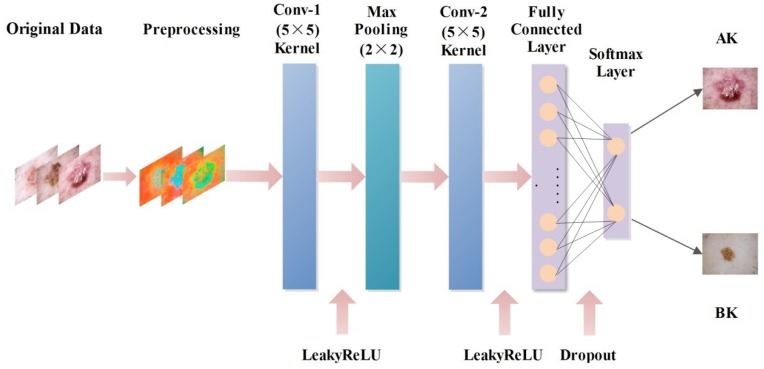
The network architecture of the actinic keratosis deep learning (AK-DL) model.

The activation function of traditional CNN models is the sigmoid function, which is prone to having the vanishing gradient problem and easily leads to failure because of its soft saturation. Instead, the ReLU function is often employed in CNN models as the activation function, which could alleviate the vanishing gradient problem. Compared with the sigmoid function, it is not necessary to calculate the exponent for the ReLU function when calculating the activation value, so it is faster than the sigmoid function. However, the ReLU function is “fragile” in the training process when applied at a rapid learning rate, resulting in unsuccessful training. To solve this problem, the LeakyReLU function was finally used in the AK-DL model as the activation function. This function introduces a Leaky value into the negative half interval, and the function output has a small slope to the negative value input. After several experiments, it was found that the recognition accuracy of actinic keratosis was the highest when the Leaky value was set as 0.01. The network architecture of the AK-DL model is shown in Figure 2.

#### 2.3.2. AlexNet Transfer Learning 

The AlexNet model was proposed by Alex Krizhevsky in 2012 and won the 2012 ILSVRC competition. This model has 8 neural network layers, including 5 convolutional layers (3 convolutional layers are followed by the max pooling layer) and 3 fully connected layers, with 630 million links, 60 million parameters, and 650,000 neurons [24]. In this study, AlexNet was used for transfer learning. First, 1000 kinds of natural images from ImageNet were pretrained to get the initialization model. Then, the number of neurons in the output layer was changed to 2, and the last layer was updated. Transfer learning can alleviate overfitting caused by insufficient data and improve training efficiency.

#### 2.3.3. GoogLeNet Transfer Learning

The GoogLeNet model was the champion of 2014 ILSVRC challenge. Unlike AlexNet, GoogLeNet has a total of 22 layers. In order to avoid the problem of vanishing gradient in deep network, two losses at different depths are added into GoogLeNet to prevent gradient vanishing. In addition, GoogLeNet possesses modular unit inception, which not only increases the accuracy of classification but also greatly reduces the number of inputs. The modified inception unit was used in this study, including 9 inception modules (the GoogLeNet V1 architecture). In order to improve training efficiency, transfer learning was also applied to GoogLeNet in this study. After pretraining based on ImageNet, three layers (fully connected layer, softmax layer, and classification output layer) were added to the last three layers of the original network. Meanwhile, the learning factor of the fully connected layer was increased to make the learning rate of the new layer higher than the original layer. Finally, the last transport layer in the network (pool5-drop_7 × 7_s1) was connected to the new layer.

#### 2.3.4. ResNet Transfer Learning

The ResNet model, which was first proposed in 2015 and won first place in the classification task in the ImageNet contest, has been extensively used for medical image processing [25,26,27]. In this study, classical architecture ResNet-50 was employed for transfer learning. After pretraining 1000 kinds of natural images from ImageNet, the initialization model was obtained, and the transfer process was then completed following the method mentioned above.

### 2.4. Traditional Machine Learning

The traditional algorithms of medical image processing were also introduced and compared in order to verify the superiority of AK-DL. The Histogram of Oriented Gradient (HOG) of the image was employed as the feature vector of the classifier input. The image resolution was modified to 200 × 200, the cell size was set as 32 × 32 pixels, and each block contained 2 × 2 cells. Finally, a 900-dimension feature vector was extracted from each sample. In this study, the classical machine learning algorithm classifiers employed included support vector machine (SVM), random forest (RF), and *k*-nearest Neighbor (KNN).

### 2.5. Parameter Optimization

Four parameters, namely, Momentum, InitialLearnRate, MiniBatchSize, and L2Regularization, were optimized with the Momentum optimizer in the CNN training process. A weighted average of the movement index based on the gradient was calculated by the Momentum optimizer, which could accelerate the learning process when facing small and continuous gradients with a lot of noise. The optimized parameters are shown in Table 1. In addition, optimization of the traditional classifiers were also performed in the training process.

### 2.6. Evaluation Indexes

In this study, accuracy (Acc), sensitivity (Sens), specificity (Spec), precision (Prec), Matthews correlation coefficient (MCC), and area under the receiver operating characteristic curve (AUC) were included as evaluation indexes. The calculations for Acc, Sens, Spec, Prec, and MCC are given below. In addition, considering the impact of training time of the model, the training time was also investigated. A 10-fold cross-validation was applied in the present study, meaning that 10% of the data was chosen randomly as the test data and the rest as the training data for 10 times. The mean values of the evaluation indexes from the 10-fold validation were calculated as the final values.

(1) $$\mathrm{Acc}=\frac{TN+TP}{TN+TP+FN+FP}$$

(2) $$\mathrm{Sens}=\frac{TP}{TP+FN}$$

(3) $$\mathrm{Spec}=\frac{TN}{TN+FP}$$

(4) $$\mathrm{Prec}=\frac{TP}{TP+FP}$$

(5) $$\mathrm{MCC}=\frac{TP\times TN-FP\times FN}{\sqrt{\left(TP+FP)(TP+FN)(TN+FP)(TN+FN\right)}}$$

where *TP* (true positive) is the proportion of the positive samples correctly classified, *FP* (false positive) is the proportion of negative samples incorrectly classified, *TN* (true negative) is the proportion of the negative samples correctly classified, and *FN* (false negative) is the proportion of positive samples incorrectly classified.

## 3. Results

### 3.1. Comparison of AK-DL with Machine Learning Models

**Figure 3:**
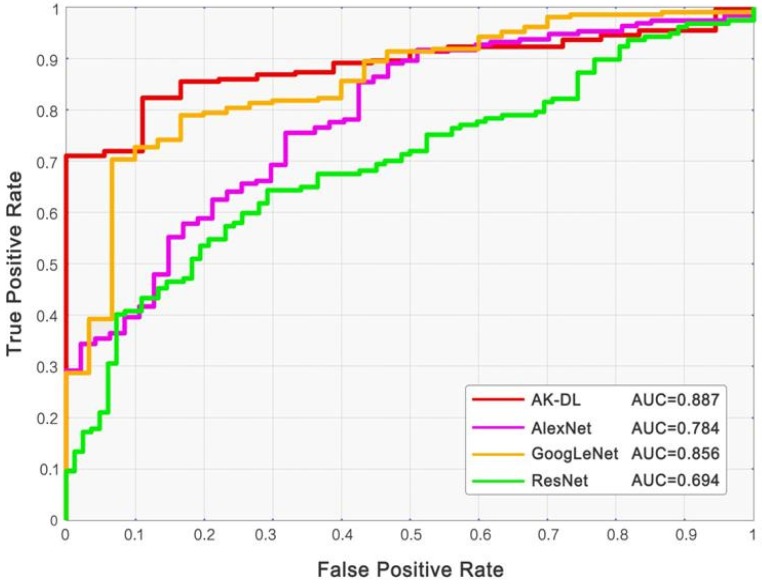
Receiver operating characteristic curves (ROC) of CNN models.

Based on the dataset of keratosis, although AK-DL had a relatively simple architecture, its performance was better than the mainstream deep CNN models, including AlexNet, GoogLeNet, and ResNet. The accuracy of AK-DL was the highest (92.5%) compared with the other models (Table 2), and its AUC was also the highest among all the CNN models (the corresponding receiver operating characteristic curves (ROC) are shown in Figure 3). It is noteworthy that the average training time of AK-DL (123.0 s) was far less than AlexNet (2426.0 s), GoogLeNet (13,761.0 s), and ResNet (15,488.0 s), suggesting that this shallow CNN model had the highest practical value in commercialization, and it was thus possible to develop a convenient and rapid diagnostic system of keratosis.

**Figure 4:**
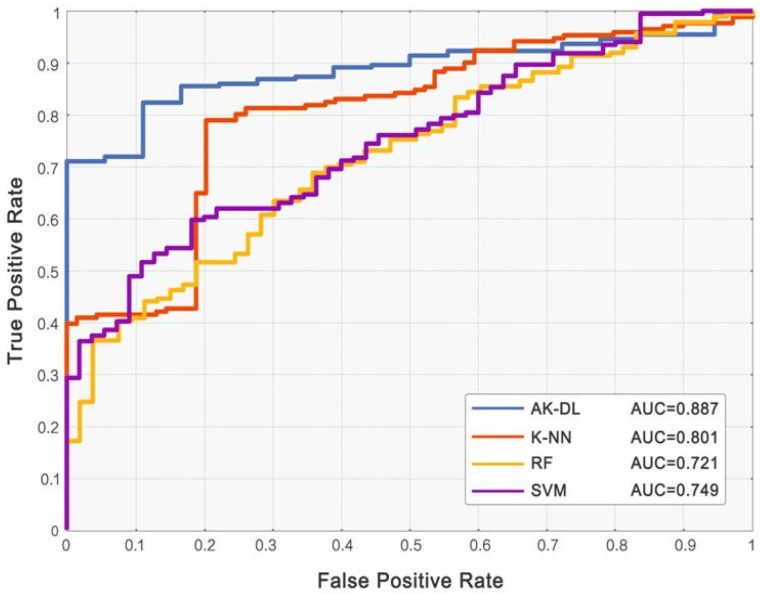
ROC curves of AK-DL and traditional machine learning algorithms.

We also calculated the evaluation indexes of traditional processing algorithms and compared them with the results of the AK-DL model (shown in Table 3 and Figure 4). Although SVM had the best classification performance among the traditional algorithms with an accuracy of 79.1% and AUC of 0.749, the AK-DL still had obvious advantages according to its higher accuracy and AUC. Remarkably, the training time of the traditional processing algorithms was less than the AK-DL model due to the simplicity of the model architecture.

### 3.2. Intelligent Diagnostic System

**Figure 5:**
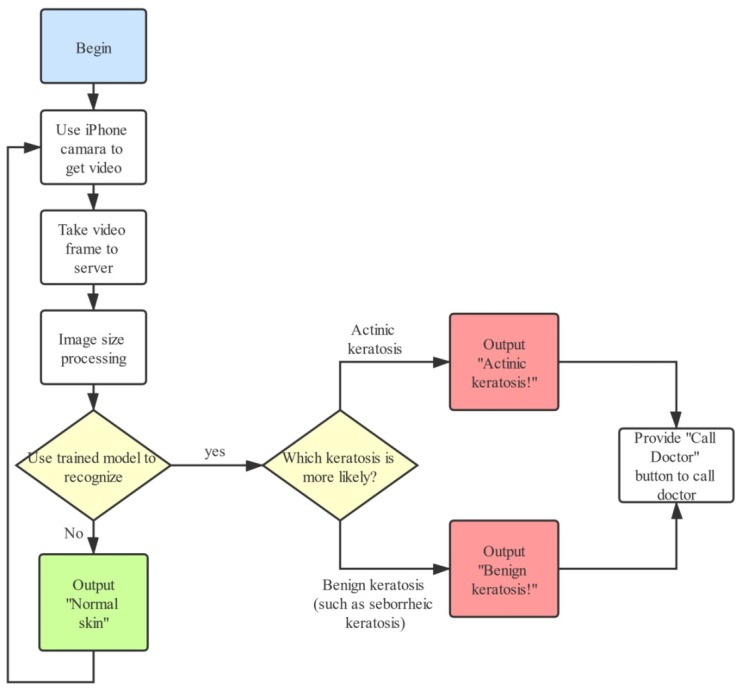
Flow chart of intelligent diagnostic system of keratosis.

**Figure 6:**
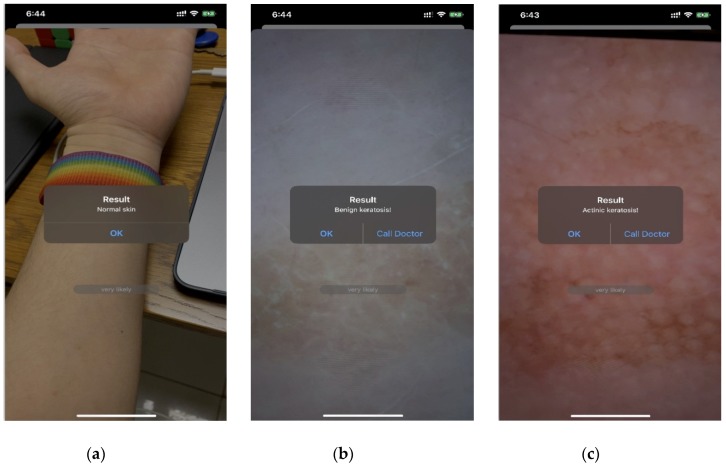
Diagnosis system interface of keratosis built on iOS. (**a**) Normal skin, (**b**) benign keratosis, (**c**) actinic keratosis.

To achieve the end-to-end mode of the keratosis diagnostic system and to make it convenient for people to easily examine their skin condition in real time, we developed an intelligent diagnostic system on iOS using the AK-DL model. The development language was objective-C, and the iOS SDK was used to build the development environment. The workflow of the system is as follows (Figure 5). Firstly, the video input stream from the iPhone camera is obtained, and representative frames are then extracted and sent to the server. Secondly, the server employs PHP to execute Python commands, and the obtained frames are sent to the trained AK-DL model via tube (the AK-DL model requires the input format to be RGB image, and all images are scaled to the unified image size of 64 × 64 × 3). Thirdly, the server transforms the background into binary data and sends it to the trained model. Finally, the output value predicted by the model will feed back to the users. The diagnostic interface is shown in Figure 6.

## 4. Discussion

In this study, a new shallow CNN named AK-DL was designed to recognize AK instead of adopting mainstream CNN architecture. The network depth was compressed in the AK-DL model to shorten training time, and specific choices in optimizer, activation function, and other aspects could improve the performance of the model. In order to prevent overfitting caused by insufficient and unbalanced image data, data augmentation and dropout layer were used in this study. Our results showed that this network possessed an outstanding performance in recognizing AK.

As there are few related studies on using deep learning algorithms to diagnose AK, we compared the results of our study with some latest studies of other skin diseases. Li et al. [28] used the deep CNN Inception-V3 for training and then made a classification test on 2000 melanoma mirror images, resulting in a final AUC value of 0.8000. Similarly, Codella et al. [29] combined deep CNNs, sparse coding, and SVM to identify melanoma and some atypical lesions and made a cross-validation on 2624 data samples, eventually obtaining an accuracy of 0.7390, a sensitivity of 0.7380, and a specificity of 0.7430. However, not all deep CNNs perform well in the recognition of skin diseases. Mirunalini et al. [30] employed GoogLeNet transfer learning based on a melanoma dataset of 2000 samples from ISIC, and only an overall accuracy of 0.6580 was obtained on the validation set. It is worth noting that the AK-DL model, a shallow CNN, was more effective than the abovementioned studies based on deep CNNs, with the accuracy reaching up to 0.925, while the sensitivity, specificity, and AUC were 0.938, 0.909, and 0.887, respectively. A comparison of AK-DL with the current mainstream deep CNNs, such as AlexNet, GoogLeNet V1, and ResNet-50, based on the same dataset of skin keratosis also reflected the superior performance of AK-DL. Theoretically speaking, deep CNN can extract more feature information for recognition. However, the textural features of skin keratosis are relatively simpler compared with the pathological images of breast cancer, lung cancer, colorectal cancer, etc. It is possible that, based on the feature extraction of special images with sample texture, the capacity of shallow CNN is not lower, and may be even higher, than deep neural networks. In contrast, considering the fact that deep CNN models have more convolution layers, the characteristics extracted are more complex and prone to overfitting when the image dataset is relatively small.

Additionally, we compared the result of AK-DL with other studies using traditional machine learning algorithms, and it also supported the superiority of the AK-DL model. Vasconcelos et al. [31] proposed a new color evaluation method and then applied feature selection and machine learning classification, resulting in an identification accuracy of 0.7775 and 0.8138 on two smaller public datasets, respectively. Ohki et al. [32] used the RF model, a traditional classifier, to automatically classify 1148 skin images (including 980 melanocytic nevi and 168 melanomas) and acquired sensitivity of 0.7980 and specificity of 0.8070 after taking a 10-fold cross-validation. It is worth noting that the performance of AK-DL, a shallow CNN, was better than the abovementioned traditional machine learning methods. This might be due to the inherent shortcomings of traditional machine learning, including the dependence of manual feature extraction and the incomplete reflection of data. On the contrary, the convolution layer was employed in AK-DL to extract features, which could extract image details with end-to-end recognition patterns.

It is worth noting that the efficiency of the AK-DL model (123.0 s for training time) was significantly lower than the deep neural networks, including the AlexNet (2426.0 s), GoogLeNet (13,761.0 s), and ResNet (15,488.0 s) models. Considering the fact that a high recognition accuracy (0.925) was achieved, our results show that shallow CNNs, such as AK-DL, can reduce the training time as well as ensure effectiveness of the model, thus contributing to a reduction in training costs. Therefore, it is possible to develop this real-time image recognition technique with high-accuracy as a supplementary diagnostic method for skin keratosis. It should be pointed out that the AK-DL model was only confirmed in the keratosis dataset, and the application of a shallow CNN thus needs more research and further exploration.

Actinic keratosis is prone to occur in sun-exposed areas in middle-aged and elderly people. It is a type of precancerous skin lesion caused by sun damage. If not actively treated, it may turn into squamous cell carcinoma [33,34]. According to statistics, the prevalence rate of actinic keratosis in Europe, the United States, and Australia has reached 4.5% to 60.0% [35]; therefore, early and active interventions should be advocated. In many cases, this disease is easily confused with BK, which is easily ignored, and diagnosis needs to be observed through pathological examination with a relatively complex process. In view of the situation above, it is of great significance to develop a portable app for accurate automatic diagnosis of AK. A portable app would not only provide real-time monitoring, such as in family doctor services for middle-aged and elderly groups, but also help complete the task of clinical diagnosis with high efficiency and low cost. Based on this, we applied our own AK-DL model to complete the development of the software and tried to put it into service. The AK-DL model performed best in the practical application of this app due to its high diagnostic accuracy and low training cost. In future, we will continue to improve the app service functions and actively promote it gradually.

In order to highlight the characteristics of deep learning for AK diagnosis, we only selected the mature and mainstream technologies for comparison. Actually, several novel methods have recently been developed to diagnose AK. Spectral diagnosis of skin precancerous lesions possesses noninvasive characteristics, which draws conclusions by analyzing and summarizing the spectroscopy information of skin components [36,37]. Compared with neural networks, this method is more accurate, although its analysis is more complicated. Additionally, employing the Markov chain model [38] or traditional statistical methods based on the high-throughput sequencing data of human skin microbial community is also one of the methods for diagnosing AK, but the cost is obviously much higher. Moreover, several other technologies, including Antera 3D technology [39,40], in-vivo or ex-vivo reflectance confocal microscopy (RCM) [41,42], and dermoscopy analysis [43], have been employed for the diagnosis of skin diseases and monitor them after treatment. The detection effect of these abovementioned methods have been verified in actual application, which are considered relatively effective, but their equipment cost and operation complexity are higher than the model proposed in our study. The requirements of equipment for the AK-DL model are relatively lower and the equipment operates simply, which provides the possibility of portable diagnosis.

## 5. Conclusions

Considering the complexity of diagnosis and the lack of attention during the early stage of AK, a shallow CNN named AK-DL was designed to promote the rapid recognition of AK with high accuracy. Our findings showed that the AK-DL model had the best performance in recognition of AK compared with mainstream deep CNNs (AlexNet, GoogLeNet, and ResNet) and traditional machine learning algorithms (SVM, RF, and KNN). Based on the AK-DL model, an intelligent diagnosis system of AK was developed to satisfy real-time and portable diagnosis. The results of the present study indicate that it is better to employ a shallow CNN in the recognition of AK, and further studies are warranted to determine whether the application of a shallow CNN may promote real-time and automatic diagnosis of other skin diseases. One limitation of this study is that we only validated the detection performance of AK-DL on the images of AK and BK. In real-life diagnosis of skin diseases, only a fraction of precancerous skin lesions are AK. Thus, we would not be able to detect precancerous skin in areas not yet visibly affected by AK due to the lack of relevant training images. In order to carry this work into real clinical setting, a large-scale database that covers different precancerous skin lesions must be built to further train and evaluate the different algorithms.

## Figures and Tables

**Table 1:** Convolutional neural network (CNN) parameters.

Parameters	AK-DL	AlexNet Transfer	GoogLeNet Transfer	ResNet Transfer
Momentum	0.9	0.6	0.9	0.7
InitialLearnRate	0.0001	0.0001	0.0001	0.0001
MiniBatchSize	10	20	15	20
L2Regularization	0.0005	0.0001	0.0001	0.00001

**Table 2:** CNN model comparison.

CNN Models	Acc	Sens	Spec	Prec	MCC	Training Time (s)
AK-DL	0.925	0.938	0.909	0.924	0.848	123.0
AlexNet	0.862	0.908	0.815	0.832	0.727	2426.0
GoogLeNet	0.874	0.874	0.875	0.901	0.746	13,761.0
ResNet	0.774	0.829	0.721	0.740	0.553	15,488.0

**Table 3:** Comparison of AK-DL and traditional machine learning algorithms.

Models	Acc	Sens	Spec	Prec	MCC	Training Time (s)
AK-DL	0.925	0.938	0.909	0.924	0.848	123.0
HOG+K-NN	0.713	0.954	0.511	0.619	0.506	12.4
HOG+RF	0.778	0.796	0.763	0.735	0.557	20.1
HOG+SVM	0.791	0.780	0.800	0.766	0.579	20.2

## Supplementary Materials

Supplementary materials can be found at https://www.mdpi.com/2075-4418/10/4/217/s1.

## References

[1] Lebwohl M. (2003). Actinic keratosis: Epidemiology and progression to squamous cell carcinoma. Br. J. Dermatol..

[2] Cantisani C., Paolino G., Melis M., Faina V., Romaniello F., Didona D., Cardone M., Calvieri S. (2016). Actinic Keratosis Pathogenesis Update and New Patents. Recent Pat. Inflamm. Allergy Drug Discov..

[3] Jetter N., Chandan N., Wang S., Tsoukas M. (2018). Field Cancerization Therapies for Management of Actinic Keratosis: A Narrative Review. Am. J. Clin. Dermatol..

[4] Lanoue J., Chen C., Goldenberg G. (2016). Actinic keratosis as a marker of field cancerization in excision specimens of cutaneous malignancies. Cutis.

[5] Didona D., Paolino G., Bottoni U., Cantisani C. (2018). Non Melanoma Skin Cancer Pathogenesis Overview. Biomedicines.

[6] Braun R.P., Ludwig S., Marghoob A.A. (2017). Differential Diagnosis of Seborrheic Keratosis: Clinical and Dermoscopic Features. J. Drugs Dermatol. JDD.

[7] Quaedvlieg P., Tirsi E., Thissen M., Krekels G. (2006). Actinic keratosis: How to differentiate the good from the bad ones?. Eur. J. Dermatol..

[8] Ortonne J.P. (2002). From actinic keratosis to squamous cell carcinoma. Br. J. Dermatol..

[9] Lebwohl M., Swanson N., Anderson L.L., Melgaard A., Xu Z., Berman B. (2012). Ingenol mebutate gel for actinic keratosis. N. Engl. J. Med..

[10] Sirinukunwattana K., Raza S.E.A., Tsang Y.-W., Snead D.R., Cree I.A., Rajpoot N.M. (2016). Locality sensitive deep learning for detection and classification of nuclei in routine colon cancer histology images. IEEE Trans. Med. Imaging.

[11] Albarqouni S., Baur C., Achilles F., Belagiannis V., Demirci S., Navab N. (2016). Aggnet: Deep learning from crowds for mitosis detection in breast cancer histology images. IEEE Trans. Med. Imaging.

[12] Li W., Li J., Sarma K.V., Ho K.C., Shen S., Knudsen B.S., Gertych A., Arnold C.W. (2018). Path R-CNN for prostate cancer diagnosis and gleason grading of histological images. IEEE Trans. Med. Imaging.

[13] Jalal Deen K., Ganesan R., Merline A. (2017). Fuzzy-C-means clustering based segmentation and CNN-classification for accurate segmentation of lung nodules. Asian Pac. J. Cancer Prev. APJCP.

[14] Gao F., Wu T., Li J., Zheng B., Ruan L., Shang D., Patel B. (2018). SD-CNN: A shallow-deep CNN for improved breast cancer diagnosis. Comput. Med. Imaging Graph..

[15] Zhang J., Hu H., Chen S., Huang Y., Guan Q. Cancer cells detection in phase-contrast microscopy images based on Faster R-CNN. Proceedings of the 2016 9th International Symposium on Computational Intelligence and Design (ISCID).

[16] Kawahara J., Hamarneh G. Multi-resolution-tract CNN with hybrid pretrained and skin-lesion trained layers. Proceedings of the International Workshop on Machine Learning in Medical Imaging.

[17] Kaur P., Dana K.J., Cula G.O., Mack M.C. Hybrid deep learning for reflectance confocal microscopy skin images. Proceedings of the 2016 23rd International Conference on Pattern Recognition (ICPR).

[18] Lopez A.R., Giro-i-Nieto X., Burdick J., Marques O. Skin lesion classification from dermoscopic images using deep learning techniques. Proceedings of the 2017 13th IASTED International Conference on Biomedical Engineering (BioMed).

[19] Harangi B. (2017). Skin lesion detection based on an ensemble of deep convolutional neural network. arXiv.

[20] Tajbakhsh N., Suzuki K. (2017). Comparing two classes of end-to-end machine-learning models in lung nodule detection and classification: MTANNs vs. CNNs. Pattern Recognit..

[21] Zhao X., Liu L., Qi S., Teng Y., Li J., Qian W. (2018). Agile convolutional neural network for pulmonary nodule classification using CT images. Int. J. Comput. Assist. Radiol. Surg..

[22] Tschandl P., Rosendahl C., Kittler H. (2018). The HAM10000 dataset, a large collection of multi-source dermatoscopic images of common pigmented skin lesions. Sci. Data.

[23] LeCun Y., Bottou L., Bengio Y., Haffner P. (1998). Gradient-based learning applied to document recognition. Proc. IEEE.

[24] Krizhevsky A., Sutskever I., Hinton G.E. Imagenet classification with deep convolutional neural networks. Proceedings of the Advances in Neural Information Processing Systems.

[25] Han S.S., Lim W., Kim M.S., Park I., Park G.H., Chang S.E. (2018). Interpretation of the Outputs of a Deep Learning Model Trained with a Skin Cancer Dataset. J. Investig. Dermatol..

[26] Wu E., Wu K., Cox D., Lotter W. (2018). Conditional infilling GANs for data augmentation in mammogram classification. Image Analysis for Moving Organ, Breast, and Thoracic Images.

[27] Mendes D.B., da Silva N.C. (2018). Skin lesions classification using convolutional neural networks in clinical images. arXiv.

[28] Li Y., Shen L. (2018). Skin lesion analysis towards melanoma detection using deep learning network. Sensors.

[29] Codella N., Cai J., Abedini M., Garnavi R., Halpern A., Smith J.R. Deep learning, sparse coding, and SVM for melanoma recognition in dermoscopy images. Proceedings of the International workshop on machine learning in medical imaging.

[30] Mirunalini P., Chandrabose A., Gokul V., Jaisakthi S. (2017). Deep learning for skin lesion classification. arXiv.

[31] Vasconcelos M.J.M., Rosado L., Ferreira M. A new color assessment methodology using cluster-based features for skin lesion analysis. Proceedings of the 2015 38th International Convention on Information and Communication Technology, Electronics and Microelectronics (MIPRO).

[32] Ohki K., Celebi M.E., Schaefer G., Iyatomi H. Building of readable decision trees for automated melanoma discrimination. Proceedings of the International Symposium on Visual Computing.

[33] Lober B.A., Lober C.W. (2000). Actinic keratosis is squamous cell carcinoma. South. Med. J..

[34] Dinehart S.M., Nelson-Adesokan P., Cockerell C., Russell S., Brown R. (1997). Metastatic cutaneous squamous cell carcinoma derived from actinic keratosis. Cancer.

[35] Zhao Y., Li C., Wen C., Wei Y., Li R., Wang G., Tu P. (2016). The prevalence of actinic keratosis in patients visiting dermatologists in two hospitals in China. Br. J. Dermatol..

[36] Gagniuc P.A., Ionescu-Tirgoviste C., Gagniuc E., Militaru M., Nwabudike L.C., Pavaloiu B.I., Vasilăţeanu A., Goga N., Drăgoi G., Popescu I. (2020). Spectral forecast: A general purpose prediction model as an alternative to classical neural networks. Chaos Interdiscip. J. Nonlinear Sci..

[37] Lanzanò L., Scordino A., Privitera S., Tudisco S., Musumeci F. (2007). Spectral analysis of Delayed Luminescence from human skin as a possible non-invasive diagnostic tool. Eur. Biophys. J..

[38] Gagniuc P.A. (2017). Markov Chains: From Theory to Implementation and Experimentation.

[39] Cantisani C., Paolino G., Pellacani G., Didona D., Scarno M., Faina V., Gobello T., Calvieri S. (2016). MAL daylight photodynamic therapy for actinic keratosis: Clinical and imaging evaluation by 3D camera. Int. J. Mol. Sci..

[40] Cantisani C., Paolino G., Corsetti P., Bottoni U., Didona D., Calvieri S. (2015). Evaluation of Ingenol mebutate efficacy for the treatment of actinic keratosis with Antera 3D camera. Eur. Rev. Med. Pharm. Sci..

[41] Mazzella C., Greco V., Costa C., Scalvenzi M., Russo D., Savastano R., Staibano S., Fabbrocini G. (2018). Management of clinical and subclinical actinic keratoses with histological and immunohistochemical assessments by confocal microscopy. Dermatol. Ther..

[42] Mercuri S.R., Rizzo N., Bellinzona F., Pampena R., Brianti P., Moffa G., Colombo F.L., Bearzi P., Longo C., Paolino G. (2019). Digital ex-vivo confocal imaging for fast Mohs surgery in nonmelanoma skin cancers: An emerging technique in dermatologic surgery. Dermatol. Ther..

[43] Carbone A., Ferrari A., Paolino G., Buccini P., De Simone P., Eibenschutz L., Piemonte P., Silipo V., Sperduti I., Catricalà C. (2017). Lentigo maligna of the face: A quantitative simple method to identify individual patient risk probability on dermoscopy. Australas. J. Dermatol..

